# Psychiatric and Psychological Implications of Congenital Heart Disease

**DOI:** 10.3390/jcm14093004

**Published:** 2025-04-26

**Authors:** Oliwia Grunwald, Agata Anna Sakowicz-Hriscu, Napoleon Waszkiewicz, Marcin Kożuch, Sławomir Dobrzycki

**Affiliations:** 1 Department of Invasive Cardiology, Medical University of Bialystok, Marii Sklodowskiej-Curie 24A, 15-276 Bialystok, Poland; marcinkozuch@poczta.onet.pl (M.K.); slawek_dobrzycki@yahoo.com (S.D.); 2 Department of Psychiatry, Medical University of Bialystok, Wolodyjowskiego 2, 15-272 Bialystok, Poland; napwas@wp.pl

**Keywords:** congenital heart disease (CHD), ASD, VSD, mental disorder, psychiatric comorbidity, risk factors

## Abstract

**Background/Objectives**: Congenital heart disease (CHD) is the most common birth defect worldwide, with a prevalence rate of 2.78 per 1000 births. CHD, as with any chronic illness, poses a certain risk of comorbidities. The prevalence rate of psychiatric disorders in adults suffering from CHD is as high as 12.4%, and in the pediatric CHD patient group, this figure is over 35%. **Methods**: An extensive literature search was conducted in reputable databases, such as PubMed, Scopus, and Web of Science, in the timeframe of November 2024 to March 2025. Ultimately, we selected 146 articles to be included in this review. **Results**: Depression, anxiety disorders, bipolar disorder, autism spectrum disorders, and PTSD are amongst the most frequently occurring. CHD concomitant with a mental disorder poses an increased risk of complications, worsening both cardiological and psychiatric outcomes. **Conclusions**: CHD is a multidisciplinary illness that needs to be treated with caution and screening for it should be integrated with investigations of psychiatric comorbidities, using scales such as HADS and BDI-2, while considering their moderate accuracy. Prevention, early detection, and intervention in CHDs are necessary steps in patient healthcare, not omitting patient education. The quality of life is also influenced by CHDs, as chronic heart failure has been confirmed as an independent factor in diminishing QoL levels. In addition to this, it extrapolates the need for the establishment of standardized guidelines regarding this topic.

## 1. Introduction

**Figure 1:**
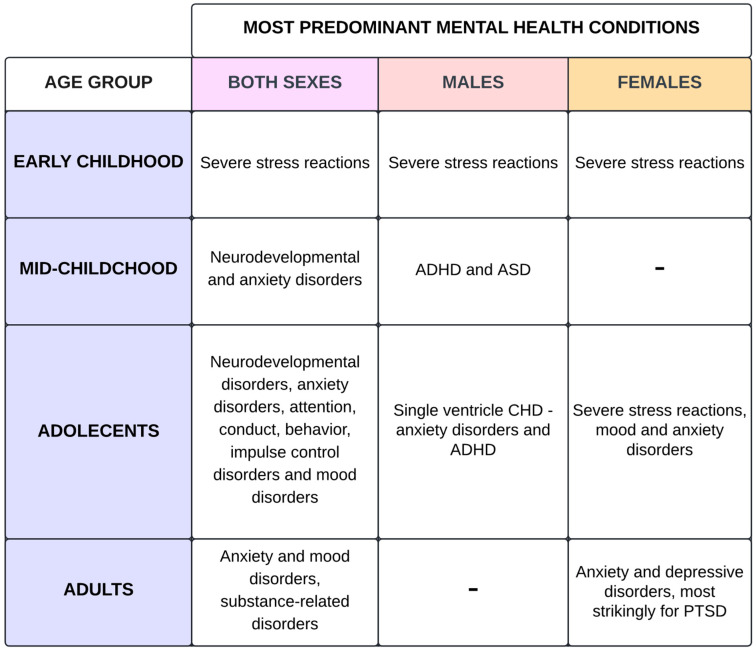
Predominant mental health conditions differentiated by age group and sex [4,11,12,15,16]. Sakowicz-Hriscu, A. Created in Lucid (https://lucid.co/, accessed on 1 January 2025).

The clinical management of congenital heart disease (CHD) is advancing due to the fact that heart defects are more common in neonates; moreover, over 97% of children with CHD are expected to reach adulthood, thus increasing the life expectancy and necessitating the supervision of cardiovascular and non-cardiovascular complications [1,2,3]. A proportion of 95.5% of CHD patients present with non-cardiac comorbidities [3]. Considering mental health disorders in individuals under 18 years of age, they prevail in 35.1%, where, in the general population, this number is 13–17% [4,5,6,7]. It is worth noting that age-related comorbidities, concomitant with CHD, develop not only in younger patients, but also have an increasing frequency in adults: approximately 6.3–12.4% of adults are diagnosed with psychiatric disorders [3,8,9]. In juxtaposition, the prevalence of mental disorders in the general adult population is around 23%, which is mostly constituted by females, young adults aged 18–25 years, and Caucasians [10]. Among the pediatric population, CHD peaks in early childhood and adolescence, during which anxiety, depression, and ADHD are the dominant mental health conditions for both sexes. In adults, the most common are depressive and anxiety disorders, alcohol/drug abuse, and PTSD, according to Figure 1 [3,4,9,11,12,13]. A proactive patient approach in CHD research should integrate genetic, phenotypical, and environmental factors and focus on quality of life, which will facilitate a more personalized medical approach [14]. The main goal of this paper is to highlight epidemiology, psychiatric burden, and risk factors in specific CHD subgroups. We aim to investigate and showcase the correlation between CHD and psychiatric outcomes, which may extrapolate to clinical patient care.

## 2. Materials and Methods

**Figure 2:**
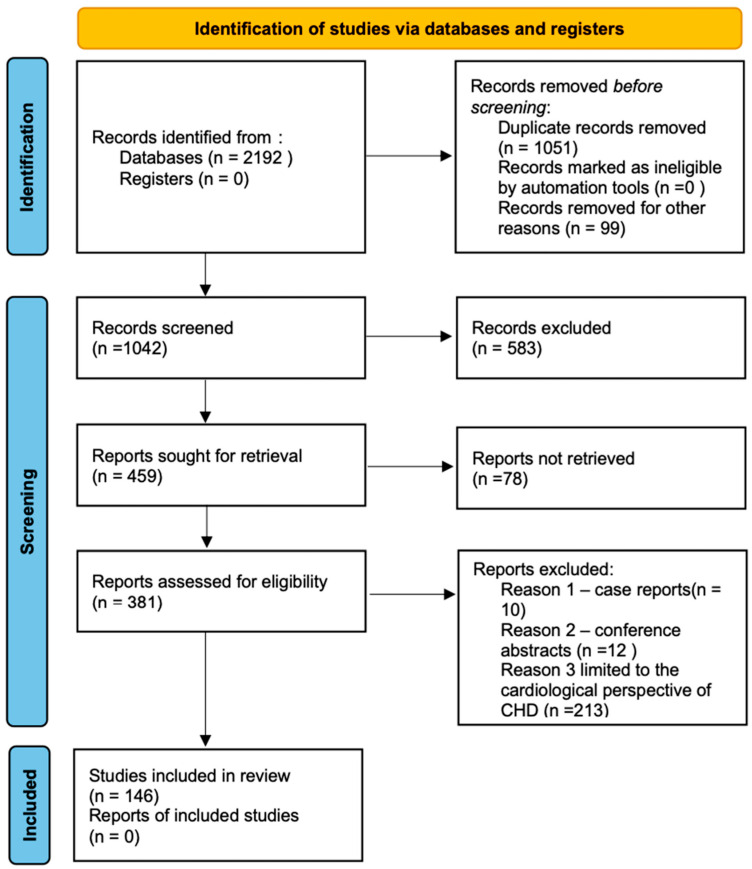
The PRISMA flowchart.

According to 2020 PRISMA reporting guidelines, a literature search was conducted on literature from November 2024 to March 2025 published in PubMed, Scopus and Web of Science databases using the following keywords: congenital heart disease, CHD, atrial septal defect, ASD, ventricular septal defect, VSD, cyanotic congenital heart disease, acyanotic congenital heart disease, mental health, mental disorder, psychiatric disorder, psychiatric disease, comorbidity, depression, anxiety, autism spectrum disorders, PTSD, ADHD, neurodevelopment, and quality of life. Also, combinations of these and similar terms were searched. Most of the literature incorporated into this review covers the timeframe of the past 15 years, with some individual papers being older, which provided references. Relevant articles, written in English and accessible online, were then included with the intention to cover the widest possible spectrum of the subject. Applicable data derived from these articles were compiled and then divided into subcategories such as CHD subgroups and domains of life. This paper provides publications from varying standpoints as to ensure a nuanced depiction of the topic at hand, concentrating on randomized clinical trials, meta-analyses, and observational studies. Information accounted for in said studies was crosschecked with similar research results on the subject in order to secure the most factual outcome. Research that was published in a language other than English or was limited to the cardiological perspective of CHD and case reports and conference abstracts were excluded. Given that the review was a collaborative effort by two independent reviewers, the risk of bias was mitigated by establishing a defined focus area beforehand to ensure a cohesive approach. In the end, 146 articles were selected (Figure 2).

## 3. Results

### 3.1. Pediatric Population

**Figure 3:**
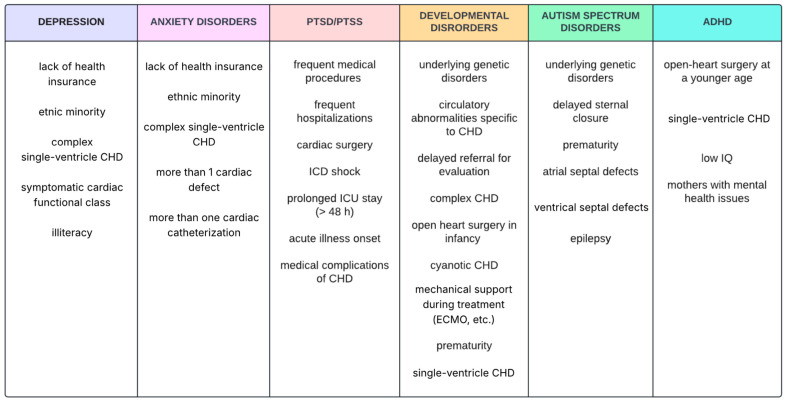
Risk factors for specific psychiatric disorders in children and adolescents with CHDs [15,18,19,20,24,28,29,30]. Sakowicz-Hriscu, A. Created in Lucid (https://lucid.co/, accessed on 1 January 2025).

The population-level analysis showcased that simple CHDs have increasing trends, such as the development of more comorbidities in patients with at least one coexisting chronic illness, in particular, genetic syndromes, i.e., trisomy 21,Turner syndrome, Marfan syndrome, and Noonan syndrome [17,18]. In total, 18.2% of the CHD pediatric patients were diagnosed with anxiety or depression, in contrast to 5.2% of those without CHD. Aside from this, the risk of anxiety and/or depression in the simple CHD subgroup of 4-to-9-year-olds amounted to 16% and was about 5 times higher in those without CHD, whereas, in complex single-ventricle CHD, the risk rose up to 7 times [11]. The pediatric population with CHD is susceptible to autism spectrum disorder, ADHD, developmental disorder/disability or developmental delay, which significantly influences the future functioning of the individual [19,20,21]. As mentioned in Figure 3, where the risk factors for the specific psychiatric disorders are presented, ADHD symptoms at school age could be more frequent in children who have undergone surgical repair of CHD in their first year of life [21]. As an ADHD characteristic, inattention prevails in patients with cyanotic heart disease or single-ventricle defect, although hyperactivity may be observed in complex CHDs and both are 3 to 4 times more frequent than in the general population [22,23]. Moreover, adolescents with single-ventricle CHDs, after undergoing the Fontane procedure, were seen to have an impaired psychosocial status and anxiety and exhibit disruptive behavior and depressive symptoms [15].

Stressful events, such as surgical and non-surgical procedures and follow-ups on a regular basis, can result in acute stress, post-traumatic stress (PTS) symptoms in 12–14% and PTSD in 12–31% of cardiac surgery subgroups [24]. Behavioral internalizing disorder in adolescents, after surgical repair of CHD, was inevitably connected with past medical history, i.e., a higher number of cardiac surgeries, an older age at the time of cardiac surgery, deep hypothermic circulatory arrest, a short gestational age, and reduced SpO2 levels [25]. Furthermore, internalization disorder rates in children and adolescents were correlated to the rate of health problems and lower physical activity, which influenced their future health conditions and quality of life factor [26,27]. Underdiagnosis was influenced by a lack of insurance or identifying as an ethnic or racial minority [11].

### 3.2. Adult Population

**Figure 4:**
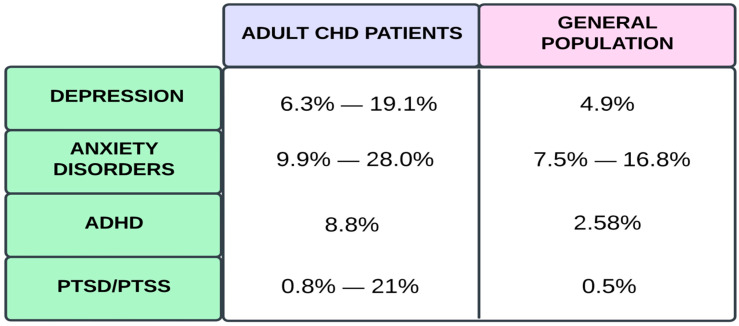
Prevalence rates of chosen psychiatric disorders amongst adult patients with CHD compared to general population [9,12,33,34,40,41,42]. Sakowicz-Hriscu, A. Created in Lucid (https://lucid.co/, accessed on 1 January 2025).

Among adults with CHD, 12–30.1% were diagnosed with or medicated for anxiety disorders, including post-traumatic stress disorder (PTSD) or depressive disorders [12,31]. The risk is about 30–50% higher than that for the general population without CHDs [12]. Comparison between the prevalence rates of specific psychiatric disorders amongst adult CHD patients and the general population is presented in Figure 4. Also, female patients with genetic syndromes and two or more cardiac procedures during a 3-year period in adolescence and adulthood had an elevated probability of a mental health disorder diagnosis [16,32]. Number of medical interventions and time interval between surgeries causes a certain level of mental distress; hence, these factors are linked specifically to post-traumatic stress symptoms (PTSS) and PTSD. It is worth noting that the sample size in two of the referenced PTSD studies were significantly smaller than that in the third one [12,33,34]. Disease-specific PTSD was predominantly associated with symptoms of depression and cardiac surgery before one year of age [12,34]. PTSD patients developed various coping mechanisms, i.e., avoidance and re-experiencing as well as hyperarousal, owing to chronic uncertainty, which may be influenced by disinformation, insufficient information, unpredictable outcomes, and patient–healthcare relation [35]. In referral to PTSD, increased and differentiated incidence ratios among researchers may be caused by complex cardiac care in privately insured patients or detection bias due to a lack of health insurance [12,36].

In view of the ESC (European Society of Cardiology) 2020 guidelines, even though the psychiatric and mental aspects of CHDs are not deliberated on, it is stated that adult CHD at a more advanced age accelerated aging, encompassed cognitive decline and sensory alterations, and diminished psychosocial functions [37]. However, adults with CHD above 60 years of age are reported to have fewer anxiety symptoms and a better emotional state than their younger counterparts. This leads to the conclusion that anxiety alternates over time in patients with CHD and its course is not consistent, according to various researchers [38].

**Figure 5:**
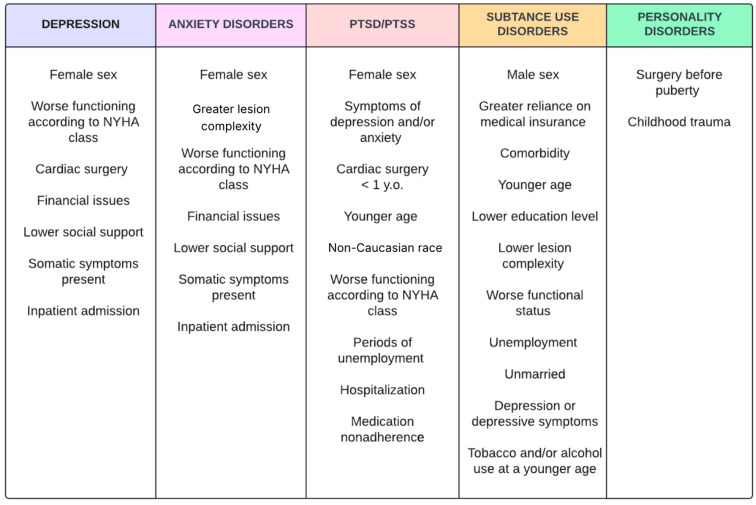
Risk factors of specific psychiatric disorders in adults with CHDs [12,16,33,43,44,45,46,47,48]. Sakowicz-Hriscu, A. Created in Lucid (https://lucid.co/, accessed on 1 January 2025).

According to 2018 AHA/ACC (American Heart Association/The American College of Cardiology) guidelines, patients with ACHD (Adult Congenital Heart Disease) should be assessed for depression and anxiety and be wary of potential neurodevelopmental impairment, which are to be sought after, due to underdiagnosis in children [39]. The predictors of depression and anxiety in CHDs were loneliness and fear of negative evaluation, though they were not affected by illness severity or functional status. Additionally, the patient-perceived health status enhanced depressive symptoms, as well as other specific risk factors mentioned in Figure 5 [13]. Contrary to that, major depression in the other study group was associated with severely complex CHD, cyanosis, worse functioning, and antidepressant therapy. It prevailed in 19.1% of CHDs and was linked to the elevated ratio of adverse effects and impaired functional status. Increased levels of hsCRP (high-sensitivity C-reactive protein) and NT-proBNPs were observed and were possibly indicative of systemic inflammation and heart failure in CHDs with major depressive episodes [40]. In total, 30.1% of the patients with CHD had elevated HADS-A and HADS-D scores, while measures of quality of life (QoL) and health status (HS) declined [31]. In screening scales, such as HADS and BDI-2, using lower cut-off levels is recommended and their accuracy is moderate [9]. Having taken the above information into account, the diagnosis of depression and anxiety may be challenging in adult CHD patients and psychiatric consultation is encouraged.

### 3.3. Quality of Life in Congenital Heart Disease

Congenital heart diseases impact quality of life (QoL), mental health, and physical function in patients, in view of prolonged life expectancy. Mainly, it is reported that QoL in CHD patients is high and it can be even better than in healthy peers when measured as life satisfaction. When QoL is measured in terms of physical functioning, patients with complex CHD perform worse than patients with moderate or mild defects or healthy individuals. The best outcomes of QoL were reported in patients with coarctation of the aorta and isolated aortic valve disease; however, the worst outcomes were reported in those with cyanotic heart disease and Eisenmenger syndrome. Functional status seems to be the paramount impact factor for QoL, which itself is connected with mood, anxiety, and substance use disorders, such as nicotine and alcohol abuse [9,49].

Patient-related determinants of low QoL were considered to be NYHA functional class, older age, unemployment, and being unmarried. The complexity of CHD, sex, and educational status were of minimal or no importance regarding the QoL [50,51]. On the other hand, it is stated that, when physical functioning is measured as QoL, complex CHD patients underperform rather than patients with moderate or mild defects or otherwise healthy individuals [52]. Furthermore, the risk of suicide and tendency to deliberate self-harm is not increased in comparison to the reference cohort in the long term [50]. Nonetheless, patients with chronic cardiovascular disease, such as chronic heart failure, pose an independent risk to psychiatric illness and progressing suicide death rates, as well as to more prevalent psychiatric disorders in CHD, causing higher hospital admission rates and longer durations of hospitalization and more frequent referral to other facilities and home healthcare [32,53,54]. Taking this into account, in adults with CHD, the health status is said to be influenced by the healthcare system. The higher the physician and nurse density, the better the QoL, physical, and mental health status and the lower the psychological distress [55]. Yet, globally, QoL is not differentiated by country of origin or healthcare system variables [52]. Chinese CHD adolescents (from 12 to 18 years of age) in comparison with the healthy population presented with a lower quality of life factor, which was enforced by disease severity, depression, the self-esteem level, and management of the CHD [56]. It is difficult to establish whether CHD is the primary cause of decreased QoL levels in CHD patients since there are multiple factors that are interlinked and layer on each other. However, chronic heart failure due to CHD undisputedly lowers QoL.

### 3.4. Social Life in Congenital Heart Disease

Congenital or acquired cardiac disease in children greatly influences familial relationships, which, in most cases, were strengthened. One-fifth of family members negatively assessed the impact on family life [57]. In parents of infants and toddlers with CHDs, there was an increased level of parental stress, sleep disturbance, anxiety, and difficulty coping [58]. Importantly, this study had a limited number of participants and included self-reporting, which could possibly influence its objectivity [57,58]. Up to 30% of parents of children with CHDs undergoing cardiac surgery in the immediate time period display symptoms of post-traumatic stress disorders, 25% to 50% display depression and/or anxiety, and 80% display severe psychological distress [59]. Parent-focused psychological aid improved the aforementioned symptoms [60]. Infants with CHD, who were hospitalized in an cardiac intensive care unit, could benefit from a protocol for nurses—Individualized Family-Centered Developmental Care (IFDC) nursing—which engages parents and enhances neurodevelopment of the children [61].

The psychological aspect of CHDs in adolescents was remarkable for lower self-esteem and more depressive symptoms [56]. High self-esteem is a crucial feature for development in adolescents to promote maturation and prevent loneliness [62]. In patients with chronic illnesses, lower self-esteem determines greater symptom severity [63]. In adolescents, more than half of complex CHDs required educational assistance [23].

Following adulthood with CHDs and coexisting psychological distress, there was a major unemployment rate, inadequate higher education, and lower quality of life [64]. One’s social life may therefore be strongly impacted by these factors—CHD patients might feel dissatisfied by social interactions due to their own limitations, as stated above. Educating patients on their affliction can possibly aid in resolving this issue, making them more confident and open.

## 4. Neurodevelopment in Congenital Heart Disease

Congenital heart defects, especially cyanotic and complex ones, can severely impact the child’s development, both mentally as well as physically. Lack of proper blood oxygenation (before and after birth), frequent medical procedures requiring mechanical support, stress, and medication are only some of the factors influencing the course of a child’s maturing. Defects that cause cyanosis tend to have a higher percentage of patients suffering from brain lesions, varying from 34% in TGA (transposition of the great arteries) to 49% in left-heart CHDs, although some studies show that it is the other way around and acyanotic CHDs have higher rates of neurologic complications [65,66,67]. This inconsistency may stem from differences in the tools used—data vary depending on the type of literature available; for example, meta-analyses report a lower percentage of prevalence compared to observational reviews [65]. Decreased MCA (middle cerebral artery) blood flow in fetuses with HLHS (hypoplastic left-heart syndrome) might be one of the causes for the previously mentioned CNS trauma, as their cerebrovascular resistance is lowered [68]. Similarly, TGA fetuses also show a trend towards cerebral vasodilation [69]. Additionally, slowed third-trimester brain growth has been reported in neuroimaging in utero [65].

From 8% to 33% of neonates requiring surgical care for CHDs (depending on the type) have microcephaly, half of which will exhibit some kind of neurobehavioral pathology such as hypo- or hypertonia, motor asymmetry, or jitteriness [19,67]. Motor, cognitive, adaptive, speech, and behavioral issues arise [70]. These can be observed as soon as 1 year of age in children who have undergone cardiac operations, and more so in those who have undergone palliative surgery unlike those who have had corrective surgery instead [71]. Leukomalacia has also been found to be more common in patients post-operation, with an incidence rate of 48%, compared to 16% before surgery [72,73], which might have to do with prolonged hypoxia in the early post-operative period [74]. However, brain injury post-cardiac surgery was shown to usually be short-term and any neurological delays present were mostly attributed to preoperative inefficient hemodynamics [75].

Although these abnormalities can be mild or not properly visualized by certain imagining methods, it has been noted that they can persist in adolescence, as CHD patients tend to have smaller total brain volumes (cyanotic CHD sufferers have an overall higher total brain volume loss) and white and gray matter volumes, with no changes in CSF volumes. These pathologies may contribute to the inhibition of language learning skills, cognition, and executive function [76,77,78]. Children with CHD usually have IQ levels within a normal range; however, they tend to fall 5 to 10 points below those of their healthy peers’, posing an additional intellectual challenge, as shown by the CHD subgroup presenting with overall lower GAI (general ability index), VAI (vocabulary acquisition index), and VCI (verbal comprehension index) scores [79,80]. Fine motor skills, short-term and working memory, and attention are also often impaired [81,82,83].

**Figure 6:**
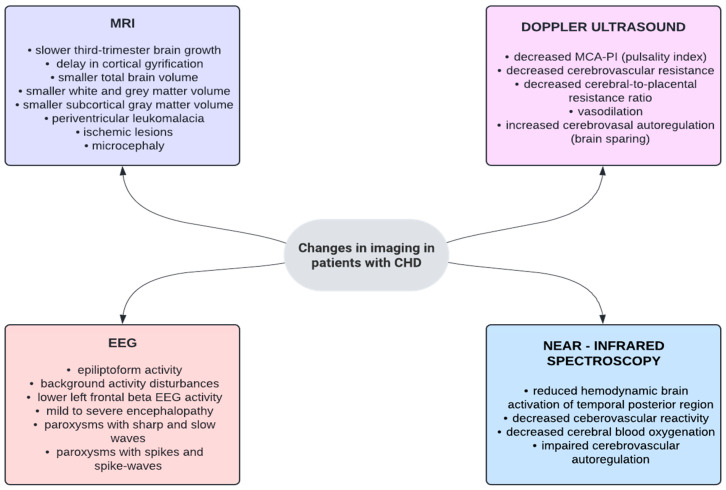
Observable neuroimaging changes in CHD [68,72,73,74,79,88,89,90,91,92,93,94,95,96]. Sakowicz-Hriscu, A. Sakowicz-Hriscu, A. Created in Lucid (https://lucid.co/, accessed on 1 January 2025).

There is evidence that the severity of the CHD positively correlates to the prevalence and level of neurological impairment—over half of all patients with complex CHD suffer from it, while mild to moderate CHDs do not exhibit such high numbers [84]. Additionally, fetuses with single-ventricle CHDs (HLHS), TGA, and TOF had much smaller head circumferences compared to those of healthy fetuses, although it was found that the general CHD group, encompassing all major CHD subtypes, did not vary much in terms of this measurement [85]. This shows that certain CHD conditions tend to carry a higher risk when it comes to cerebral malmaturation, followed by numerous neurobehavioral challenges. This can be seen in the genetic aberrations that are frequently associated with CHD, such as 22q11.2 deletion or trisomy 21, since they often coexist with other mutations; in DiGeorge’s syndrome, genes partaking in both heart and brain formation, namely, TBX1 and HIRA, are compromised [86]. Genetic material pathologies are rarely selective and more often than not alter organisms on an organic and functional level. In syndromic CHD, a staggering 90% of patients struggle with some kind of neurodevelopmental disability, although it is clear that, in these cases, they are more of a result of the underlying genetic disorder than solely of the CHD itself [84]. Nevertheless, it is crucial to stay vigilant and screen for possible neurological impairment using available tools. As Figure 6 demonstrates, EEG might be beneficial for patients who have suspected encephalopathy or epilepsy, MRI proves useful in early neuroimaging (especially preoperatively) for further neurological prognosis, and Doppler USG can possibly help visualize any cerebrovascular complications of CHD. Being able to observe any neurological changes in childhood might have clinical applications further in the patient’s life and constitutes an area for future research.

In conclusion, CHDs affect children’s nervous system in various ways and often result in neurodevelopmental disabilities, making prevention, early detection, and multidisciplinary intervention crucial elements of CHD care. Modifiable medical risk factors and concrete strategies need to be established in order to influence the overall risk of such neurological abnormalities and improve the long-term neurofunctional outcome [87].

### 4.1. Ventricular Septal Defects

Ventricular septal defects (VSDs) are the most common congenital cardiac anomalies found in children and the second most common in adults (the first being bicuspid aortic valve). As of April 2024, according to the National Library of Medicine, VSDs account for 37% of all congenital heart defects in children, this number being lower in adults, as VSDs tend to spontaneously close in around 90% of these cases [97]. VSDs have been linked to some long-term cardiological complications, such as arrhythmias, pulmonary hypertension, and Eisenmenger syndrome, among many others; however, its link to certain mental disorders has not been so widely explored. VSDs can produce a wide spectrum of hemodynamic instabilities, ranging from desaturation to long-term left-to-right shunt, which, in turn, may greatly impact one’s quality of life and lead to depression or anxiety. Patients with isolated VSDs are at a higher risk of mood affective disorders compared to healthy individuals and have higher use of antidepressants and anxiolytic drugs. They also display a lower work participation, especially those who additionally suffer from a psychiatric disorder, which further impacts their social wellbeing. According to the same study, the most common psychiatric problem in patients with VSDs, however, are emotional disorders (ICD F40-F48) such as phobias, anxiety disorders, and OCD, which occur in around 33.8 per 10,000 VSD patients [98]. This shows that people suffering from clinically relevant VSDs are in danger of loneliness and social alienation as well as bearing additional costs due to being prescribed anxiolytic medication. This all can possibly lead to further worsening of their mental status.

A lower education level was also observed in this particular group, with a larger percentage of patients with VSDs having basic education as the highest level of education compared to the control group, putting them at further risk of mental stress, as a low education level is listed as one of the major risk factors for many psychiatric illnesses. VSD patients are also at a higher risk of ADHD, with inattention being the leading symptom in most of the participants. Similarly, it has been noted that it is more common for these patients not to seek tertiary education, having secondary or primary education as the highest level of schooling [41]. This might be linked to the fact that intellectual disabilities were significantly more prevalent in VSD sufferers, with an incidence rate of 8.5 per 10,000, whereas said rate was only 2.2 per 10,000 in controls [98]. This is the biggest discrepancy when it comes to the overall burden of psychiatric disorders between patients with this cardiac anomaly and a healthy control group. It is also worth noting that patients with known chromosomal abnormalities did not participate in this cohort study; therefore, said intellectual disabilities are most likely not a result of such aberrations and the reason for their frequency in VSD patients lies elsewhere. Such a reason might be that a certain percentage of patients with VSDs need to undergo corrective cardiac surgery in infancy, which entails the usage of deep hypothermic circulatory arrest (DHCA). Its longer duration was correlated to worse physical endurance, which, in turn, was linked to lower self-esteem in these children as well as poor school competence, most likely as a result of neurodevelopmental dysfunction. However, these issues did not specifically impact the quality of life of families whose children underwent the procedure, interestingly, not even when the child was suffering from a cyanotic heart defect (such as tetralogy of Fallot) and was subject to prolonged preoperative hypoxemia [99].

### 4.2. Cyanotic Congenital Heart Defects

Tetralogy of Fallot, Ebstein anomaly, hypoplastic heart syndrome, and truncus arteriosus are among the more widely recognized cyanotic heart defects leading to less efficient circulation in the lungs and low blood oxygen. This, in turn, causes a multitude of changes in the workings of other organs, including the brain. It is well known that insufficient oxygen supply to the central nervous system not only negatively impacts its functions but also causes irreversible structural damage if chronic [100,101]. Neurodevelopment in children with CHD is therefore often affected by these organic changes contributing to impairment of learning, communication, and motor skills [102]. However, this might be remedied to an extent by surgically repairing the defect [103], although some argue that it is the other way around and surgical intervention (both cardiac and non-cardiac) is associated with increased risk of mental impairment and psychiatric disorders in these patients [104]. While the aforementioned issues are somewhat fixable or preventable in children, this is not the case for adults, since the CNS, once fully developed, loses a lot of its plasticity. Adult patients with cyanotic CHD are far more likely to experience a cerebrovascular event, which may impact their cognitive functions, adding to the psychological stress they already might have been experiencing since childhood [105].

Children with cyanotic heart defects demonstrated more symptoms of anxiety than those with acyanotic ones [106]. Similarly, adolescents who underwent surgical correction of cyanotic CHD are reported to have significantly higher post-traumatic stress symptom (PTSS) scores and are slightly more likely to suffer from anxiety disorders [107]. Cardiac surgery before puberty has been correlated to a higher rate of personality disorders as well, where a staggering 75% of patients diagnosed with PD (personality disorder) and CHD underwent said surgery at a young age [108]. All of this results in the worrying prevalence of mental health problems in CHD patients and more frequent psychiatric hospitalization [16,101,109,110,111].

Depression has also been found to correlate to the likeliness of cyanosis, where 12,1% of depressed CHD patients had cyanosis versus 5.7% in nondepressed CHD patients, as well as increased HF (heart failure) biomarkers, heightening the overall mortality risk even more and worsening the prognosis [40,112]. Quality of life is impacted by these factors, seeming to be on an overall lower level in pediatric patients with cyanotic CHD (compared to both acyanotic CHD patients and healthy controls), lowering even further in those who have had medical intervention, including surgery [113]. This often continues into adulthood, with studies about adult CHD sufferers reporting similar findings: their QoL is usually significantly diminished, especially in the cyanotic CHD group and paradoxically in those who have had the defect operatively cured [114]. Curiously, however, even though, overall, psychiatric comorbidities are more frequent and QoL is lowered in this group of patients, the suicide rate in this group is comparable to that of healthy individuals. Not only that, but suicide occurs later in life for these people and there are not any major differences observed when it comes to suicide rates and the complexity of the lesions, meaning cyanotic CHD patients did not have an overall higher risk of suicide [50].

It is worth noting that all of the aforementioned problems impact not only the patient themselves but their carers too. A proportion of 1% of children in the US are born with a heart defect every year, 25% of them with critical ones requiring one or more surgeries in their very first year of life [59]. In 2021, in Poland, around 56 per 100,000 newborns died due to a congenital disorder, of which 42 per 100,000 were cardiac defects [115]. It is unsurprising that the parents of these children are reported to have an increased risk of PTSD, anxiety disorder, and depression, sometimes as high as 4-fold when it comes to mothers [59]. A higher incidence of depression and anxiety were found in mothers of children suffering from cyanotic CHD compared to those whose children had acyanotic CHD [116]. Younger parents (below 29 years of age) of CHD patients are also more prone to irritability and aggression than their older counterparts [115]. Taking the above stated problems into account, parents of these children are very susceptible to psychological stress of varying intensity, which, in turn, will most likely also affect their offspring, who are already at risk of mental health issues. It is essential to therefore underline that doctors treating cardiac malformations need to be more vigilant when it comes to psychiatric disabilities, not only with their patients, but their guardians as well. Early intervention might be greatly beneficial to bettering the child’s and not neglecting the parents’ mental and physical status as well as counteracting any possible future implications that prolonged emotional stress and other psychological issues arising from frequent hospital visits, surgery, and comorbidity may have.

### 4.3. Atrial Septal Defect

Atrial septal defects (ASDs) occur in around 1.6 per 1000 children and are the most frequently underdiagnosed form of CHD in childhood [117]. A fairly notable increase in the ASD prevalence has been observed in the past 50 years, although it is associated with improved imaging methods rather than the incidence of the anomaly itself. This type of CHD is often asymptomatic, which paradoxically might add to the stress of being diagnosed with it, since patients usually do not suspect any complications.

**Figure 7:**
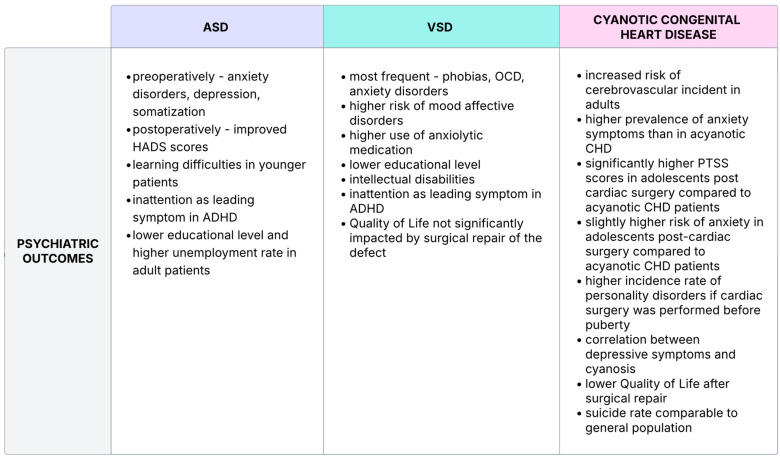
Correlation between CHD types and psychiatric outcomes [16,40,41,50,59,97,98,99,100,101,102,103,104,105,106,107,108,109,110,111,112,113,114,115,116,117,118,119,120,121,122,123]. Sakowicz-Hriscu, A. Created in Lucid (https://lucid.co/, accessed on 1 January 2025).

ASD patients are said to have an elevated long-term risk of psychiatric and developmental disorders, thus leading to the use of psychotropic medications [118,119]. Small unrepaired atrial septal defects (ASDs) were associated with predominant symptoms of anxiety, depression, and somatization. Not only surgical correction of the congenital defect but also being diagnosed with an ASD under 15 years of age posed an increased risk of the aforementioned. Transcatheter intervention for ASDs seems to be positively correlated with the quality of life [118]. There was a connection made between ASDs and educational attainment. More patients had only a high-school-level education than a university or college diploma [120]. This may be related to extrapolated ADHD symptoms, both introvert and extrovert, which may impact academic achievements. Inattention, rather than hyperactivity, may be the leading cause and put demand on special teaching techniques during school age [119]. Mild cognitive dysfunction, especially after the correction of ASDII, involves visuospatial skills, language, attention, verbal memory, executive function, and social perception, which impact school performance [119,121]. About one-fourth of an ASD patient group at 30 years of age was unemployed, and was also diagnosed with a psychiatric disorder, which contributed to being a permanent social security beneficiary [122]. All in all, follow-up on a regular basis is a warranted approach, both in operated and non-operated ASDs [121]. Percutaneous closure of ASDs betters QoL outcomes. Medical professionals and guardians ought to pay attention to any learning difficulties and lower school performance in younger CHD patients, as it might impact future job perspectives. Adults struggling with job retention due to their affliction should seek counseling and could possibly benefit from group therapy to avoid alienation while unemployed. Figure 7 summarizes the aforementioned psychiatric burden findings in different CHD types.

## 5. Future Directions

The lack of official and structured guidelines calls for the formation of them. These can only be established if future research on the topic is conducted. Investigations on how cardiac surgery in both acyanotic and cyanotic pediatric patients impacts their mental status are needed. A study touching on this subject is underway, including QoL outcomes of neonatal and infant TOF repair [42]. Additionally, prospective and retrospective studies ought to be performed on the subject of neuroimaging changes in more age groups to track the evolution of such anomalies and their clinical implications. The currently available data are limited and some of them are contradictory at times; therefore, it is challenging to make informed statements and suggestions in certain aspects of the area. The Neuro-moms study aims to explore the impact of paternal or the co-parent’s mental health on one-year olds with CHD [124]. Transcatheter ASD occlusion follow-up research is ongoing, taking into consideration mental impairment as one of its adverse effects [125]. VSD percutaneous closure (PERI-CLOSE) takes on measuring an overall success rate of the procedure, encompassing functional measures of QoL such as NYHA class [126].

## 6. Discussion

Congenital heart disease, as any chronic illness, poses an increased risk of mental health deterioration due to emotional and physical stress; however, it is crucial to note that many factors impact the frequency of psychiatric comorbidities and not all of these can be accounted for while estimating such risk nor have all of them been included in this article. Moreover, each patient needs to be assessed individually in order to ensure the most accurate results. Even when the above stated conditions are met, it is essential to remember that certain tools such as surveys, scales, and questionnaires used in both psychiatry and cardiology have limited use and their scores do not often precisely portray factual results, which is dictated by a lack of standardization, imprecise point systems, and the subjective nature of these instruments, especially in psychiatry, where the presence and intensity of symptoms is problematic to measure objectively. The lack of follow-up by both patients and doctors also influences the data and one may argue that the real rate of prevalence of psychological issues among the CHD group is much higher or lower since many patients do not report back, doctors do not screen for non-cardiological comorbidities or a definitive psychiatric diagnosis is never established. Additionally, certain groups of people suffering from CHD are more prone to one type of psychiatric disorder than others; however, the risk factors for these in the CHD patient group often overlap with the risk factors presented for general population, meaning that it is sometimes difficult to access with certainty whether the actual overall risk is statistically higher for CHD sufferers.

Difficulties also arise with pediatric CHD subgroup screening, as children are rarely as elaborate in describing their emotional state as adults are and that only refers to those who are old and mature enough to discuss their mental wellbeing. Infants and children diagnosed with severe intellectual disabilities or neurodevelopmental abnormalities do not often possess the means to inform their guardians and doctors of their exact mental state; therefore, it is very challenging to screen them for particular types of symptoms. This may be why it seems that psychiatric disorders stemming from organic pathologies are more prevalent in these children than those originating from chemical imbalances, e.g., depression or anxiety disorders. It is also necessary to mind the fact that it is the parents who are responsible for providing their children with medical care and follow-up.

## 7. Conclusions

It is vital to extrapolate the need for psychiatric and psychological screening in the congenital heart disease population, both in adults and children. Outlined scientific statements should be applied in clinical settings and promote interdisciplinary solutions for an increased quality of life and psychological comfort. Chronic heart failure is known to be an independent risk factor for lowering QoL in CHD patients; however, we are not aware of any other confirmed factors. Such medical complications arising from the cardiac pathology and mental health comorbidities associated with it are interlinked, worsening both the cardiological and psychiatric outcomes. Prevention, early detection, and intervention are essential points in clinical CHD care, as psychiatric comorbidities among this group of patients are prevalent. Using neuroimaging tools like EEG, MRI, Doppler USG, and near-infrared spectroscopy in infants and children has proven to be valuable in early neurological investigations. It is encouraged to actively utilize such instruments and apply them clinically, as they can be beneficial for future neurodevelopmental prognosis. It is also recommended to consult any findings with a specialist to ensure the most objective and informed approach. In child and adolescent screening for ADHD, learning disabilities, PTSD/PTSS, and affective disorders can help in early symptom detection and prevent serious psychiatric complications. Similarly, in adults, anxiety disorders, depression, PTSD/PTSS, substance use disorders, and ADHD are to be considered as possible comorbidities. To achieve the best and most credible results, the use of standardized screening scales such as HADS and BDI-2 is proposed, keeping in mind lower cut-off levels and other limitations. Collaboration with psychiatric specialists should be established beforehand to ensure the most effective care.

## Data Availability

No new data were created or analyzed in this study.

## Abbreviations

The following abbreviations are used in this manuscript:

ADHD	Attention-deficit/hyperactivity disorder
ASD	Autism spectrum disorder
PTSD	Post-traumatic stress disorder
CHD	Congenital heart disease
ICD	Implantable cardioverter-defibrillator
ICU	Intensive care unit
ECMO	Extracorporeal membrane oxygenation
PTSS	Post-traumatic stress syndrome
NYHA	New York Heart Association
MRI	Magnetic resonance imaging
EEG	Electroencephalography
MCA-PI	Middle cerebral artery pulsatility index
